# Editorial: Combat sports and well-being: prevention, protection, and development across the lifespan, volume II

**DOI:** 10.3389/fpsyg.2026.1776952

**Published:** 2026-01-15

**Authors:** Simone Ciaccioni, Flavia Guidotti, Elena Pocecco, Nemanja Stankovic, Pascal Izzicupo

**Affiliations:** 1 Department of Education and Sport Sciences, Pegaso Telematic University, Naples, Italy; 2 Department of Movement, Human and Health Sciences, University of Rome “Foro Italico”, Rome, Italy; 3 Department of Human Sciences and Promotion of Quality of Life, University of Rome “San Raffaele”, Rome, Italy; 4 Department of Sport Science, University of Innsbruck, Innsbruck, Austria; 5 Faculty of Sport and Physical Education, University of Niš, Niš, Serbia; 6 Department of Medicine and Aging Sciences, University of Chieti-Pescara “Gabriele D'Annunzio”, Pescara, Italy

**Keywords:** combat sports, educational feasibility, inclusion, intergenerational judo, psychological resources, resilience, sport integrity, wellbeing

## Scope and rationale

The prevention, protection, and human development potential of combat sports and martial arts is increasingly being recognized. Building on the success of Volume I, *Combat Sports and Well-being: Advancing Health and Inclusion in Athletes and Practitioners*, this Research Topic, Volume II, gathers nine contributions that collectively advance conceptual understanding, identify possible psychosocial and neurophysiological mechanisms, and address implementation challenges across settings and age groups. In doing so, the Topic promotes a lifespan approach to wellbeing that considers safety, ethics, and the circumstances in which participation in combat sports can be beneficial rather than harmful.

## Conceptual and mechanistic foundations

A first cluster of papers strengthens the theoretical foundations needed to interpret the paradox of “controlled combat” as a practice that may prevent violence while involving techniques that could be extremely effective and therefore have the potential to cause harm outside a regulated context. In particular, the theoretical article of Barreira et al. proposed a phenomenological framework that deals with “corporal fighting” as a distinctive lived experience, differentiating it from brawling and from play-fighting. Furthermore, the study emphasizes how training may cultivate reflective and ethical engagement through affective and empathic modulations. This contribution is complemented by another theoretical paper, Gabriel, which advances an integrative, multiscale mechanistic account linking martial arts training demands (sensorimotor, cognitive, and socio-emotional) to brain health optimization. By proposing testable hypotheses and candidate markers, this paper provides a roadmap for research designs capable of moving beyond descriptive associations toward a mechanistic explanation.

## Psychological resources and subjective wellbeing in emerging adults

A second cluster focuses on psychological functioning and wellbeing outcomes in adolescents and young adults, while also clarifying plausible pathways through which these outcomes may occur. In a cross-sectional study on Chinese undergraduates, Ying and Yang reported positive associations between participation in combat sports and subjective wellbeing. Moreover, the authors modeled a chain mediation in which emotional intelligence and self-esteem partially account for this relationship. Although causal inference is necessarily limited by design, the proposed pathway is practically relevant because it identifies candidate targets for program design and evaluation, especially when combat sports are implemented as health-promoting activities in educational settings.

## Inclusive and therapeutic-oriented applications for neurodevelopment

Among other issues, the Topic provides evidence for inclusive and therapeutic-oriented applications. Using a randomized controlled trial, Fu and Shi evaluated a 24-week intervention involving structured martial arts games for children with autism spectrum disorder. The authors reported improvements in behavioral impairments and gross motor functions compared to a control group. This study simultaneously contributed to “protection” and “development” in combat sports by highlighting the importance of engaging structured, pedagogical approaches that can be integrated into special educational contexts. However, it also emphasized the need for careful program supervision and outcome monitoring in vulnerable populations.

## Emotion regulation and resilience: the role of instructors

Two papers have examined the psychosocial resources that may buffer distress and support wellbeing in combat sport communities across adulthood. Through qualitative, empirical-phenomenological research involving masters in Brazil, Portugal, and Spain, Santos et al. described how disruptive situations are experienced and managed. The authors focused on emotional control, resilience, and the pedagogical/ethical role of instructors in maintaining the boundary between regulated combat and harmful escalation. Complementarily, de Lorenco-Lima et al. used quantitative modeling with Brazilian jiu-jitsu athletes to show that variables such as aggression, self-control, life satisfaction, and resilience are associated with mental health indicators, with some sex-specific patterns emerging. Together, these studies highlight the importance of instructor practices, community norms, and individual self-regulatory resources in preventing and protecting against mental health issues, particularly within the context of broader mental health promotion agendas in combat sports.

## Promoting intergenerational participation and healthy aging

At later life stages, the Topic emphasizes the importance of social connection and inclusive participation in determining wellbeing. As part of the Erasmus+ Sport JOY Project, Perazzetti et al. surveyed judo coaches about intergenerational programs. The study identified safety-oriented priorities, perceived roles, barriers, and facilitators, and highlighted how coaching strategies can encourage empathy, a sense of belonging, and mutual respect between people of different ages. This article broadens the scope of wellbeing beyond individual outcomes to encompass relational and community-level processes, thus aligning with contemporary approaches to healthy and active aging.

## Implementation constraints and educational feasibility in schools

Implementation science and governance have emerged as additional pillar of protection. In a school setting, Xue et al. analyzed teachers' perspectives on a Chinese School Martial Arts Program. The authors identified how evaluations of difficulty, risk, effort-reward balance, and perceived educational value influenced participation modes ranging from effective engagement to resistance. The aforementioned modes are shaped by structural constraints. These findings reinforce the hypothesis that the effectiveness of martial arts curricula in education is not only determined by their content, but also by institutional support, teacher competence development, and stakeholder attitudes that influence feasibility and safety.

## Integrity and procedural justice in elite sporting environments

The Research Topic also addresses fairness and integrity in high-performance contexts, which can be conceptualized as a protective measure for athletes as well as for the sporting system. In the context of Olympic taekwondo, Zhang et al. explored the feasibility of using artificial intelligence to assist with video reviews. They reported that their dataset shows strong agreement with expert decisions and substantial reductions in review time. The paper suggests that technological support could be complementary rather than a substitute, highlighting the ongoing need for human oversight in ambiguous cases. In this sense, safeguarding wellbeing encompasses procedural justice and transparent decision-making, which can mitigate the perception of arbitrariness and the associated stressors in elite sporting environments.

## Synthesis and future directions

**Figure 1:**
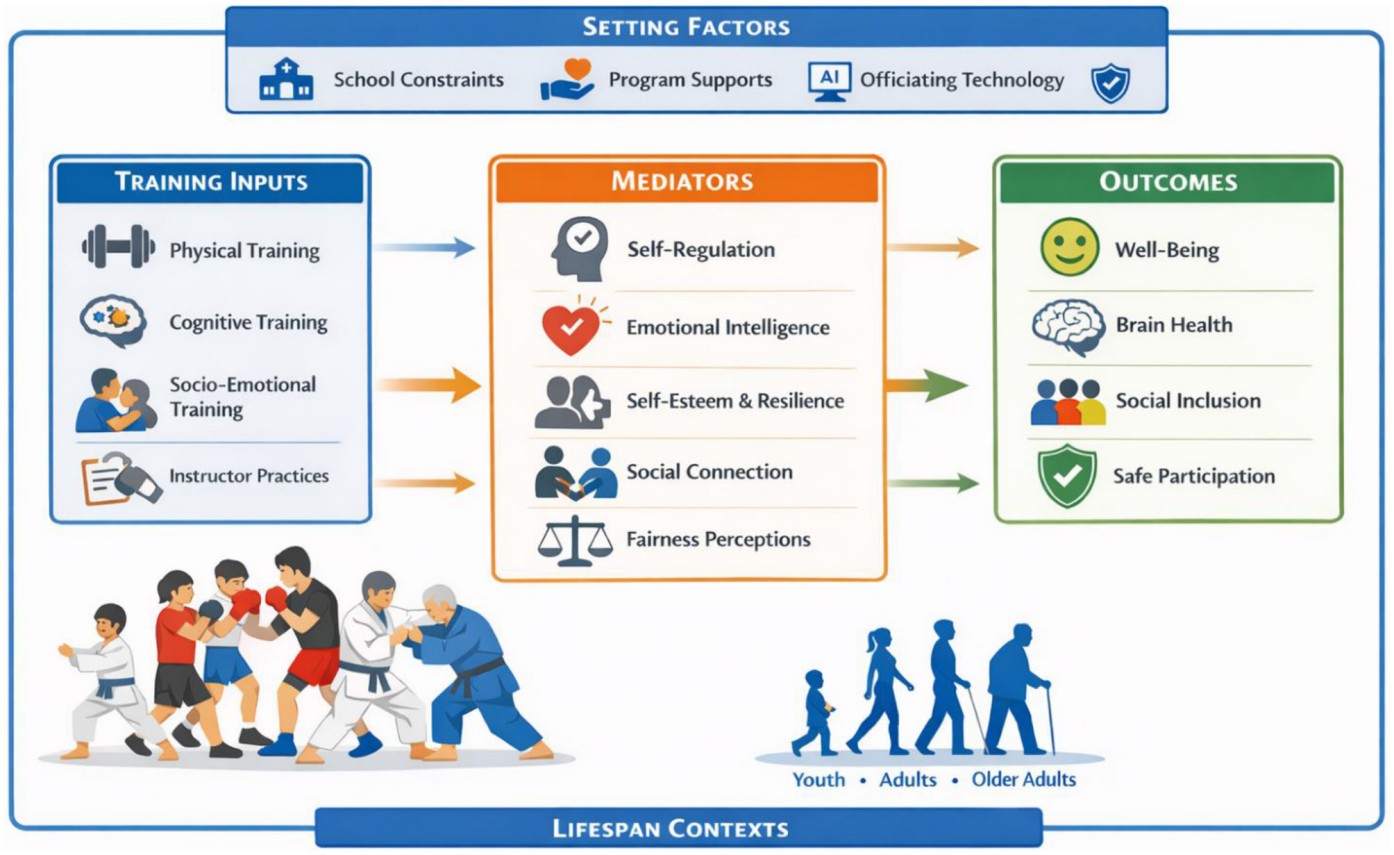
A combat sport-based lifespan framework. AI, artificial intelligence.

Taken together, the papers in Volume II show that the relationship between “combat sports” and “wellbeing” is not a singular issue, but rather a range of interconnected questions concerning mechanisms, pedagogy, implementation conditions, and governance (Figure 1). Three cross-cutting implications emerge across the papers. Firstly, achieving conceptual clarity on the nature of combat experiences is crucial for interpreting outcomes and preventing the misattribution of regulated practice to harmful aggression. Secondly, the positive effects on wellbeing appear to depend on structured training environments that explicitly cultivate self-regulation, relational ethics, and safety norms, particularly among young people and vulnerable groups. Thirdly, wider systems, including schools, sports clubs, federations, and officiating infrastructures, influence exposure to risks and access to benefits, thereby shaping prevention and protection on a larger scale.

